# Editorial: New compounds, novel targets and mechanism study in inflammation-associated liver diseases

**DOI:** 10.3389/fphar.2022.1090504

**Published:** 2022-12-09

**Authors:** Baoming Wu

**Affiliations:** School of Pharmacy, Anhui Medical University, Hefei, China

**Keywords:** liver diseases, immune cells, immunoregulation, novel targets, drug discovery

This Research Topic was initiated to investigate the pathogenesis and pathophysiological processes of inflammation-associated liver diseases, with particular focus on targets research, drug conversion, and druggability research, based on the informatics approach and further discovery of novel therapeutic targets and strategies for treatment.

Total of 18 manuscripts were submitted and reviewed, with 9 manuscripts accepted and others rejected. Scopes of accepted manuscripts involved liver injury, autoimmune hepatitis, liver ischemia/reperfusion injury and liver fibrosis. There were 3 manuscripts that discussed effects of Chinese traditional medicine on liver diseases: paeoniflorin alleviates liver injury in hypercholesterolemic rats through the ROCK/AMPK pathway (Liu et al.), mechanism of Hydroxysafflor yellow A on acute liver injury based on transcriptomics (Hou et al.), and saikosaponin d alleviates liver fibrosis by negatively regulating the ROS/NLRP3 inflammasome through activating the ERβ pathway (Zhang et al.). There were 2 manuscripts that studied the effects and mechanism of chemical medicine on liver diseases: hydroxychloroquine attenuates autoimmune hepatitis by suppressing the interaction of GRK2 with PI3K in T lymphocytes (Jiu et al.), and roxadustat, a hypoxia-inducible factor 1α activator, attenuates both long-and short-term alcohol induced alcoholic liver disease (Gao et al.). There were 3 manuscripts that revealed the effects and mechanism of cytokines on liver diseases: inhibition of macrophage migration inhibitory factor (MIF) suppresses apoptosis signal-regulating kinase 1 to protect against liver ischemia/reperfusion injury (Chen et al.), human umbilical cord blood mononuclear cells ameliorate CCl4 induced acute liver injury in mice via inhibiting inflammatory responses and up-regulating interleukin-22 (Zhang et al.), and interleukin-6 receptor blockade can increase the risk of nonalcoholic fatty liver disease: indications from Mendelian randomization (Li et al.). There was 1 review manuscript in this Research Topic, which summarized the current literature regarding FAT10 role in developing liver diseases and potential therapeutic targets for nonalcoholic/alcoholic fatty liver disease and hepatocellular carcinoma (Wimalarathne et al.).
